# How do Pacific Island countries add up on contraception, abortion and reproductive coercion? Guidance from the Guttmacher report on investing in sexual and reproductive health

**DOI:** 10.1186/s12978-021-01122-x

**Published:** 2021-03-25

**Authors:** Angela Dawson, Alec Ekeroma, Donald Wilson, Amanda Noovao-Hill, Leeanne Panisi, Brooke Takala, Kirsten Black, Deborah Bateson

**Affiliations:** 1 The Australian Centre for Public and Population Health Research, Faculty of Health, University of Technology Sydney, Ultimo, Australia; 2 National University of Samoa, Apia, Samoa; 3 Institute of Pacific Health Research, College of Medicine, Nursing and Health Sciences, Fiji National University, Suva, Fiji; 4 Lautoka Hospital, Lautoka, Fiji; 5 College of Medicine, Nursing and Health Sciences, Fiji National University, Suva, Fiji; 6 National Referral Hospital Solomon, Honiara, Solomon Islands; 7 Marshall Islands Women’s Research Initiative, Majuro, Marshall Islands; 8 Discipline of Obstetrics, Gynaecology and Neonatology, The Sydney University Central Clinical School, Camperdown, Australia; 9 The Australian Centre for Public and Population Health Research, University of Technology Sydney, Sydney, Australia

**Keywords:** Reproductive health, Pacific Islands, Health information

## Abstract

The Guttmacher-Lancet Commission report on Sexual and Reproductive Health and Rights called for the acceleration of progress to achieve SRHR that is essential for sustainable development. To integrate the essential services defined in this report into universal health coverage in the 11 sovereign nations in the Pacific, quality data is required to ensure needs are met efficiently and equitably. However, there are no comprehensive reports for Pacific Island countries that provide insight into all areas of SRHR. We collated the latest literature to identify the most up-to-date relevant data from United Nations and Guttmacher Institute reports to discern gaps in SRHR information and services relating to contraception, abortion and reproductive coercion. Investment is urgently required to strengthen health information systems for SRHR in the Pacific.

## Background

In 2018, the Guttmacher-Lancet Commission on Sexual and Reproductive Health and Rights (SRHR) outlined an essential package of sexual and reproductive health (SRH) interventions underpinned by human rights [1]. Globally, many of these SRHR goals remain neglected in policy and planning [2]. In the Pacific, addressing these key yet sensitive SRHR areas of contraception, abortion care, and reproductive coercion is crucial to achieving progress towards the Sustainable Development Goals (SDGs). However, advancement continues to be hampered by stigma and discrimination, opposition from religious institutions, insufficient knowledge, and misinformation [3–5]. Additionally, the COVID-19 pandemic has likely exacerbated the challenges of achieving progress towards the SDGs, including the provision of universal access to SRH services and rights to ensure healthy lives and well-being (Target 3.7, SDG 3) and gender equality (Target 5.6, SDG 5). Modelling of the potential impact of the COVID-19 pandemic on SRH in low and lower-middle-income countries, where services are disrupted [6], suggests that a 10% decline in the use of short and long-acting reversible contraceptives would result in an additional 28,000 maternal deaths. Alongside worldwide extreme weather events and protracted human conflict, this pandemic has shone a spotlight on SRHR inequalities and sustainable development to address these inequalities.

Increased investment in SRH by governments to overcome disparities and meet the SDGs, as outlined by the recent Guttmacher report [2], requires evidence of country-specific gaps and challenges. This not only includes detailed data about contraception prevalence, fertility, and gender indicators but also information concerning the social-economic, legal, and political determinants of SRHR, which underlie inequity.

However, there are no comprehensive reports for Pacific Island countries that provide insight into all areas of SRHR. Available documents include data on a selection of countries [7, 8] or indicators [9]. Some reports combine data on specific groups of Pacific Island countries [10] or pool data from all countries to give a regional picture of Oceania [11]. These documents do not reveal the unique and diverse contexts of each Pacific Island country. Reports mainly focus on maternal health [12] or specific populations such as adolescents [13]. Demographic and Health surveys are only available for Samoa [14] and Multiple Indicator Cluster Surveys (MICS) for Vanuatu [15], Tonga [16] and Kiribati [17], and Samoa (preliminary findings, no dataset 2020). There is little understanding of the prevalence and outcomes of unsafe abortion. It is unclear what information is available concerning reproductive coercion that describes behaviours that interfere with a woman’s autonomous reproductive health decision-making (e.g., sabotage of contraception and pregnancy coercion or controlling pregnancy outcomes) [18].

We sought to collate available information across multiple datasets to provide an up-to-date snapshot of SRHR in Pacific Island countries. We included where possible the prevalence of and access to contraception and abortion care and reports of gender-based violence and reproductive coercion for populations groups including women, adolescents, people with disabilities and lesbian, gay, bisexual, transgender and intersex (LGBTQI) people. We provide recommendations for enhancing data collection and reporting to support Pacific Island countries to meet their SRHR SDG targets.

### The island countries of the Pacific

**Fig. 1:**
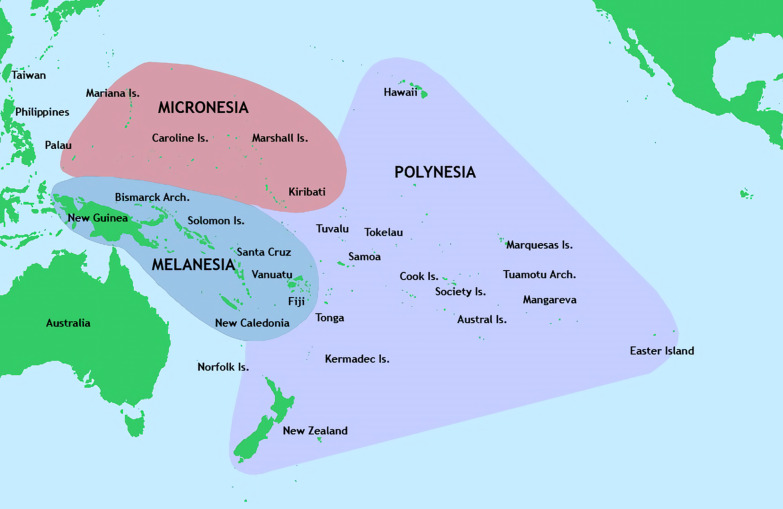
Regions of Oceania: Micronesia, Melanesia, and Polynesia [52]

There are 11 countries according to the World Bank’s Pacific Island grouping [19] with a collective population of about 2.3 million people (excluding Papua New Guinea 8.6 m). These countries lie across the Oceanic cultural regions of Polynesia*,* Melanesia*,* and Micronesia (Fig. 1)*.*

While the population of each of these 11 countries is under two-hundred thousand people and are youthful, they are culturally diverse, and most are in the upper-middle-income country (UMIC), followed by the low and lower middle-income country (LLMIC) bracket [20]. Nauru is the most densely populated of the group, while the Solomon Islands and Vanuatu, which are among the poorest, are the least populated. Palau is the only high-income country (HIC) in this grouping and has the lowest population growth rate (Table 1).

**Table 1:** Estimated population size, growth, age group and density, country size, and income

Country (last census/official estimates)	2020 population estimate	Growth rate	% of population 15–24 years	0–24 years	2018 Density	Area (km^2^)	Income group^b^
FSM (2010/2015)	115,173	1.06%	20.1	51.3	164/km^2^	700	LLMIC
Fiji (2017)	897,295	0.73%	16.4	45.4	49/km^2^	18,270	UMIC
Kiribati (2015)	119,699	1.57%	17.7	53.6	147/km	810	LLMIC
Marshall Islands (2011/2017)	59,190	0.68%	18^a^	58^a^	329/km^2^	180	UMIC
Nauru (2011/2015)	10,824	0.63%	17^a^	56^a^	541/km^2^	20	UMIC
Palau (2015/2017)	18,094	0.48%	15^a^	35^a^	39/km^2^	460	HIC
Samoa (2016/2017)	198,614	0.67%	18.1	55.3	70/km^2^	2,830	UMIC
Solomon Islands (2009/2017)	721,000	2.55%	19.1	59.1	25/km^2^	27,990	LLMIC
Tonga (2016)	105,845	1.15%	20	54.7	147/km^2^	720	UMIC
Tuvalu (2012/2016)	11,792	1.25%	19^a^	52^a^	393/km^2^	30	UMIC
Vanuatu (2016)	308,145	2.42%	18.1	56.6	25/km^2^	12,190	LLMIC

[46] ^**a**^[47]. ^**b**^[20]

## Reproductive health indicators, policy, and services

### Contraception

Research on contraception use, particularly by type, and the quality of contraceptive services is very limited in the Pacific. The SDG target 3.7 aims to ensure universal access to SRH care services, including family planning, SRH information and education, and integrating reproductive health into national strategies and programmes by 2030. Two key indicators are the: proportion of women of reproductive age (aged 15–49 years) who have their need for family planning satisfied with modern methods (Indicator 3.7.1); and the adolescent fertility rate (aged 10–14 years; 15–19 years) per 1000 women (Indicator 3.7.2). A review of intrauterine contraception across the Pacific identified that usage rates are very low across all Pacific Island countries with an overall prevalence rate of 0.3%, ranging from 0.2% in Samoa to 3.8% in Nauru [21].

As per Table 2, up-to-date projections for these key indicators are available for unmarried and married women in six countries. For the remaining states, 2015 estimates are only available for in-union women. Estimated contraceptive prevalence rates are under 50% for all countries. Alongside low contraceptive prevalence rates, very high adolescent fertility rates of around 50 births per 1000 women aged 15–19 (compared with 12 per 1,000 in Australia) are documented in seven countries: Kiribati, Marshall Islands, Nauru, Samoa Solomon Island, Vanuatu, and Fiji. These rates suggest there is a large unmet need, particularly in younger women.

**Table 2:** Abortion legality status of Pacific Island countries

Country		Abortion legality category [11]					
		Prohibited altogether (no explicit legal exception)	To save life of woman	To save life of woman/preserve physical health	To save life of woman/preserve physical health/mental health	To save life of woman/preserve physical/mental health/ on socioeconomic grounds	No restriction as to reason (with gestational and other requirements)
FSM		✓					
Fiji						✓	
Kiribati			✓				
Marshall Islands		✓					
Nauru					✓		
Palau		✓					
Samoa					✓		
Solomon Islands			✓				
Tonga		✓					
Tuvalu			✓				
Vanuatu				✓			

### Abortion

Abortion is legally restricted throughout the Pacific region but is permitted in certain circumstances in most countries, including, for example, if the pregnancy endangers a woman’s life [22, 23] (see Table 2). No national data are collected on abortion in any Pacific Island country. One estimate of prevalence suggests that between 0% and 4% of 20–24-year-olds have had an induced abortion, but this is likely to be underestimated [13]. However, in the Federated States of Micronesia, 12.8% of ever-pregnant women reported having an abortion [24]. Data from National Studies on Family Health and Safety (FHS) in some Pacific countries provide insight into induced abortion. The restricted nature of abortion likely affects women's ability to freely report whether they have undergone this procedure. None of the 634 women surveyed in the National Study on Domestic Violence against Women in Tonga reported having had an abortion [25].

Few details are available concerning abortion-related services across the Pacific. In Kiribati, none of the 15 health facilities visited as part of a UNFPA needs assessment provided information about services to prevent unsafe abortion and management of post-abortion care. Records of visits for care following unsafe abortion were not available [26].

### Reproductive coercion

The percentage of women subjected to physical and sexual intimate partner violence (IPV) in the last 12 months and their lifetime is shown in Table 3. IPV is used as a proxy measure [27] for the SDG 5.2.1 indicator: Proportion of ever-partnered women and girls aged 15 years and older subjected to physical, sexual or psychological violence by a current or former intimate partner in the previous 12 months, by a form of violence and by age.

**Table 3:** Selected reproductive indicators across 11 Pacific Countries

Country	Contraception demand satisfied by modern methods: married women 2020^a^	Contraception demand satisfied by modern methods: unmarried women 2020^a^	CPR modern all women	AFR 2015–2020 15–19 yrs	%of women subjected to physical &/or sexual IPV in the last 12 months^h^ (year of collection)	%of women subjected to physical &/or sexual IPV in their lifetime^h^ (year of collection)
FSM	–	–	49.5^c^	13.9^c^	26 (2014)	32.8 (2014)
Fiji	65.3^a^	59.7^a^	30^a^	49.4^c^	23.7 (2010)	64.1 (2010)
Kiribati	44.7^a^	72.7^a^	16.2a	51^d^	43.4 (2018)	61 (2018)
Marshall Islands	57^b^	–	43.1^b^	85^e^	20 (2014)	50.9 (2012)
Nauru	44.6^b^	–	27.5^b^	81^e^	22.1 (2013)	48.1 (2013)
Palau	55.4^b^	–	34.4^b^	29^e^	9.9 (2013)	25.2 (2013)
Samoa	37.8^a^	40.8^a^	15.9a	55^f^	22 (2000)	46.1 (2000)
Solomon Islands	55.5^a^	52.4^a^	32^a^	78^c^	41.8 (2008)	63.5 (2008)
Tonga	50.7^a^	60^a^	17^a^	30^ g^	18.9 (2009)	39.6 (2009)
Tuvalu	43.7^b^	–	27.1^b^	44^e^	25 (2007)	36.8 (2007)
Vanuatu	59.7^a^	63.1^a^	40.3^a^	49.4^c^	44 (2009)	60 (2009)

^1^Demand satisfied by modern = Women of reproductive age (aged 15–49 years) who have their need for family planning satisfied with modern methods Median = MEDIAN ESTIMATE (adjusted) projected. CPmod = Women of reproductive age (15–49 years) who are currently using any modern method of contraception Median = MEDIAN ESTIMATE (adjusted) projected [48]. ^b^Demand for family planning satisfied with modern methods among married or in-union women aged 15 to 49, 2015 Median estimate. Contraceptive prevalence (modern methods) among married or in-union women aged 15 to 49, 2015 Median estimate [49]. ^c^Age-specific fertility rates (births per 1,000 women) 15–19 years 2015–2020 [46]. ^d^[17]. ^e^Age-specific fertility rates (births per 1000 women) 15–19 years [50]. ^f^[14]. ^g^[16]. ^h^[51]

AFR, Adolescent fertility rate; CPR, Contraceptive prevalence rate; IPV, Intimate partner violence

Family Health and Safety (FHS) Studies undertaken in seven Pacific countries may shed some light on socio-cultural norms that affect a women’s ability to make autonomous decisions. Indicators include being obliged to have sex with one’s husband [24]. However, these surveys are outdated and do not include all operational definitions of violence, including those related to reproductive coercion. Exceptions are surveys from Kiribati and the Solomon Islands. These surveys indicate that 11.6% of currently partnered women in each country who had experienced physical or sexual partner violence reported that their partner had ever tried to stop family planning versus 9.3% and 7.2% respectively of those who had not experienced IPV [28]. The FHS survey from the Marshall Islands reports that 1.2% of women who experienced violence in pregnancy had abortions [29]. While in Palau, 0.8% of ever-pregnant respondents said that they have ever had an abortion and 1.7% of ever abused women reported ever having an abortion compared to 0.5% of never abused women [30].

Research in other Pacific countries provides some understanding of reproductive coercion, but these are limited by sample size and date. In a study of respondents in Fiji who have experienced physical and/or sexual partner violence, 10.2% reported that their partner had ever refused or stopped contraception, and 13.6% stated that their current or most recent partner refused to use a condom [31]. In Vanuatu, a needs assessment identified that 14–21% of women wishing to use family planning methods and 74–78% of women wanting to use condoms have either been subjected to or fearful of physical and sexual violence from their intimate partner [32].

## Discussion

This review of available data has identified gaps in SRH information and services in many Pacific Island countries. This leaves many women and other marginalised populations such as LGBTI people vulnerable to breaches of their SRHR. The Pacific Islands Forum, the region’s premier political and economic policy organisation, has recognised that significant barriers to equitable SRHR still exist and that comprehensive SDG planning, implementation and reporting cannot be achieved without access to accurate data [33, 34].

Of particular note is the widespread lack of data on contraception, abortion, and reproductive coercion amongst adolescent sexually active unmarried women and those with a disability [35]. This is due to the ethical and socio-cultural precariousness of data sampling among these groups [34]. Data collection on contraceptive indicators is essential for planning and evaluating services and determining workforce needs, and without it, a coordinated response is unattainable. The lack of abortion data is also problematic as this impedes knowledge of pregnancy outcomes and prevents an understanding of health needs. Research indicates an association between higher maternal mortality rates and unsafe abortion and restrictive abortion laws [36]. Unsafe abortion contributes to 30 maternal deaths per 100,000 live births in Oceania, higher than the 20 per 100,000 live births reported for South-eastern Asia [37]. The decriminalisation of abortion increases the proportion of safe abortions [38].

SRH data collection is also impeded by a lack of recognition of sexual diversity in many Pacific Island countries [39] and an inability to record whether a person identifies as LGBTI within existing data sets. Gaps in reproductive coercion data are also concerning as this disproportionately affects women and LGBTQI people who are also experiencing IPV and women of lower socioeconomic status [18, 40]. Besides, no data are available that document the experience of LGBTQI people and their satisfaction with SRH services. This data gap prevents an examination of health inequities that are directly linked to societal discrimination of sexual orientation, variations in sex characteristics and gender identity, as well as structural barriers that restrict access to appropriate health care.

Beyond data collection, one of the key challenges in meeting any of the SDG goals is community understanding about SRH. Several studies have highlighted the paucity of knowledge of contraception amongst women in studies in the Pacific. A cross-sectional study of 1441 women in the Solomon Islands reported that one in six pregnant women (16.95) did not know any modern contraceptive methods [41]. Another survey of 3,092 women who had given birth at the Colonial War Memorial Hospital in Fiji found that 59% (119/202) had not previously used any contraception method [42].

Statistical modelling of data from Vanuatu and the Solomon Islands has found that increasing investment in family planning would increase modern contraception from 36.8 to 65.5% in Vanuatu and 28.5 to 37.6% in the Solomon Islands by 2025. These investments have also been predicted to reduce unintended pregnancy, fertility rates, and improve maternal and infant outcomes while saving $112 million in health and education expenditure [43].

Increasing knowledge about contraception is important, but so is changing attitudes towards gender-based violence. The recent MICS surveys include data on attitudes toward domestic violence, including whether a husband is justified in beating his wife if she refuses to have sex with him [17, 16]. These surveys reveal alarmingly high rates of acceptance of violence towards women for various reasons, but data on reproductive coercion is not explicitly collected. As such, there is no information on whether partner/spousal opposition prevents women's efforts to use contraceptives or if partner/spousal abuse prevents successful use of contraceptives (e.g., contributes to contraceptive failure) [44].

## Conclusion

Investment is urgently required to improve the health information systems in Pacific countries. Capacity building is needed to support data collection to plan SRH services that can adequately respond to all populations' needs and provide a benchmark to assess service and policy quality. National routine, complete and systematic data collection on contraception, abortion, and reproductive coercion should be implemented and reported annually in each country. This will require developing a minimum data set of standardised SRH information that recognises the integrated nature of SRH is required to develop comprehensive services. Indicators such as the SDG targets and those suggested by the WHO for reproductive health programs in the Western Pacific region [45] are a useful starting point; however, they must be developed in collaboration with vulnerable populations. This partnership approach will ensure the acceptability of the proposed minimum data set and raise awareness and understanding of the importance of SRH and the demand for services. Alongside these efforts, strengthening Pacific nations' health data collection systems is essential to assess the achievement of an integrated and comprehensive rights-based approach to SRH.

## Data Availability

All data is available in the public domain.

## Abbreviations

AFR: Adolescent fertility rate
CPR: Contraceptive prevalence rate
FHS: Family Health and Safety surveys
HIC: High-income country
IPV: Intimate partner violence
LGBTQI: Lesbian, gay, bisexual, transgender and intersex people
LLMIC: Low and lower-middle-income country
MICS: Multiple Indicator Cluster Surveys
SDGs: Sustainable Development Goals
SRHS: Sexual and reproductive health and rights
WHO: World Health Organization
UMIC: Upper-middle-income country
UN: United Nations
